# Population-specific calibration and validation of an open-source bone age AI

**DOI:** 10.1038/s41598-025-20148-w

**Published:** 2025-09-23

**Authors:** Sebastian Rassmann, Luka Abashishvili, Elene Melikidze, Anastasia Sukhiashvili, Megi Lartsuliani, Ivane Chkhaidze, Nino Tskhvediani, Tinatin Gordeziani, Ekaterine Kvaratskhelia, Nino Kheladze, Maia Rekhviashvili, Salome Rodonaia, Natia Sukhitashvili, Nata Urushadze, Peter Krawitz, Tinatin Tkemaladze, Behnam Javanmardi

**Affiliations:** 1 Institute for Genomic Statistics and Bioinformatics, University Hospital Bonn, Bonn, Germany; 2 German Center for Neurodegenerative Diseases, Bonn, Germany; 3 Department of Molecular and Medical Genetics, Tbilisi State Medical University, Tbilisi, Georgia; 4 Department of Pediatrics, M. Iashvili Children’s Central Hospital, Tbilisi, Georgia; 5 Department of Pediatrics, Tbilisi State Medical University, Tbilisi, Georgia; 6 Department of Physics, Biophysics, Biomechanics and Informational Technologies, Tbilisi State Medical University, Tbilisi, Georgia; 7 Division of Genetics, Givi Zhvania Pediatric University Clinic, Tbilisi State Medical University, Tbilisi, Georgia; 8 Department of Endocrinology, American Hospital Tbilisi, Tbilisi, Georgia; 9 Department of Endocrinology, David Metreveli Medical Centre, Tbilisi, Georgia; 10 Department of Endocrinology and Pediatric Endocrinology, Ilia State University, Tbilisi, Georgia; 11 Endocrine and Diabetes Centre, M. Iashvili Children’s Central Hospital, Tbilisi, Georgia; 12 Ilia State University, Tbilisi, Georgia; 13 Department of Endocrinology, First Medical Clinic, Tbilisi, Georgia; 14 Department of Radiology, David Tatishvili Medical Centre, Tbilisi, Georgia; 15 Department of Radiology, MediClubGeorgia, Tbilisi, Georgia; 16 Department of Radiology, Georgian-Dutch Hospital, Tbilisi, Georgia

**Keywords:** Artificial intelligence, Pediatric bone age, Hand x-rays, Model calibration, Global health equity, Open-Source, Growth disorders, Bone, Skeleton, Bone imaging, Radiography, Diagnosis, Medical imaging, Paediatrics, Paediatric research

## Abstract

Assessing skeletal maturity through bone age (BA) evaluation is crucial for monitoring children’s growth and guiding treatments, such as hormonal therapy and orthopedic interventions. In recent years, artificial intelligence (AI) methods have been developed to automate BA assessment. However, bone growth patterns may vary by ancestry, and many AI models are trained on limited population datasets, raising concerns about their applicability to populations not included in the training process. To address this shortcoming for the case of the Georgian population, we retrospectively collected 381 pediatric hand X-rays and established a manual BA reference rating from seven local pediatric radiologists and endocrinologists. We then used a subset of 121 images to perform a sex-specific linear calibration of the open-source AI, *Deeplasia*, creating *Deeplasia-GE*. On the held-out test set (*n* = 260), the default version of *Deeplasia* achieved a mean absolute difference (MAD) of 6.57 months, which improved to 5.69 months after calibration. We observed that the default *Deeplasia* overestimates the BA in the Georgian cohort with a signed mean difference (SMD) of + 2.85 and + 5.35 months for females and males respectively, which after calibration is significantly reduced to -0.03 and + 0.58 months for females and males, respectively. We find that *Deeplasia-GE* has a smaller error than all the raters and, by design, *Deeplasia-GE* inherits the high test-retest reliability from *Deeplasia.* These findings suggest that *Deeplasia-GE* is a reliable AI-based BA assessment method for Georgian children.

## Introduction

The accurate assessment of bone age (BA) is crucial for assessing children’s growth and developmental progress, particularly when therapies or orthopedic interventions are considered^1^. BA can be estimated by examining the ossification centers in a child’s skeleton, particularly in the hands, wrists, and knees^1^. These centers reflect the process of bone development, where cartilage gradually ossifies and epiphyseal (growth) plates eventually fuse with the bone shafts as the child matures^1^. Among the assessed regions, the hand and wrist provide a stronger correlation with overall growth and the onset of puberty compared to the knee. Therefore, BA estimation using hand X-rays is especially effective for detecting delayed or advanced growth and is widely used as a standard diagnostic and monitoring method^1^.

Traditional methods for BA assessment, such as the Greulich-Pyle (GP^2^) and Tanner-Whitehouse (TW^3^) methods, rely on experienced clinicians’ manual interpretation of hand radiographs. However, this process is labor-intensive, subjective, and suffers from intra- and inter-rater variability, leading to inconsistencies in patient care^4,5^.

In the past decade, Artificial Intelligence (AI) techniques have shown great potential for the automation of tasks and improvement of the diagnostics processes across all medical fields^6 ^including pediatric radiology^7^ and orthopedics^8^. Various automated approaches have been introduced for BA assessment^9–13^ and have already been adopted in the clinical routine, especially in high-income countries^14–18^.

However, population bias is a critical issue in the development, validation, and application of AI in clinical settings^19–21^. Various studies have shown differences in the sex-specific growth patterns of children from different ancestries, which can influence automated BA assessment^22–26^. Thus, applying automated BA assessment methods requires careful validation and, potentially, calibration in the respective target population.

Several previous works explored the application of existing BA tools to populations (e.g. Turkish, Arab, and Korean) underrepresented or not included in the training phase of the existing BA assessment AI tools^15,17,27–29^. Some confirmed the suitability allowing for the application of these tools to the respective patients^15 ^while some works observed a deterioration of accuracy in other populations^17 ^which could hamper their applicability.

However, testing for and adapting automated BA methods to populations not included in their training requires sufficiently large cohorts with manual reference ratings. Therefore, BA tools addressing smaller populations can be unattractive, especially for commercial tools that generally prefer to target larger populations and middle to high-income countries.

**Fig. 1:**
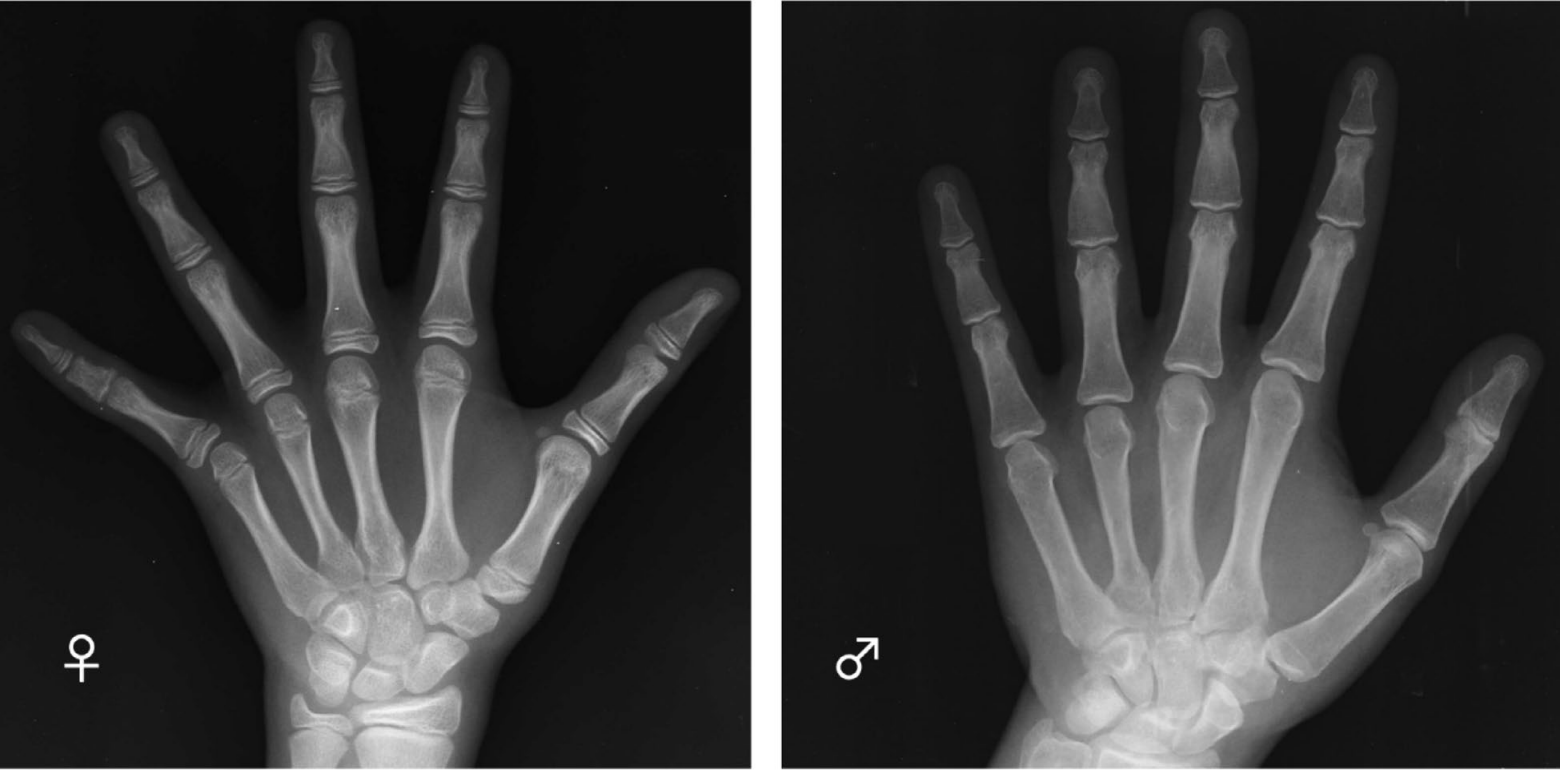
Example hand X-rays in the Georgian Bone Age Dataset. Left: A girl with chronological age of 117 months, reference bone age of 138 months, and *Deeplasia-GE* bone age of 137 months. Right: A boy with chronological age of 213 months, reference bone age of 214 months, and *Deeplasia-GE* bone age of 216 months.

In this work, we aim to address the lack of automated BA assessment for the Georgian population. To our knowledge, no automated BA tool has been tested on a Georgian population. *Deeplasia* is a state-of-the-art open-source BA assessment tool^5^ which was trained using the 2017 pediatric BA challenge of the Radiological Society of North America (RSNA) collected from two US hospitals^11,12^. It was shown to generalize to four large ethnicities within the US and German children, including those with skeletal dysplasias. Moreover, *Deeplasia* showed high test-retest precision and, thus, suitability for longitudinal applications^5^. Here, we calibrated and tested *Deeplasia* for BA assessment of children and adolescents living in Georgia by assembling a large cohort (*n* = 381) of Georgian children on which seven local clinicians conducted reference BA grading. We name this the Georgian Bone Age Dataset (GBAD). Two example hand X-rays from this dataset are shown in Fig. 1.

## Results

**Fig. 2:**
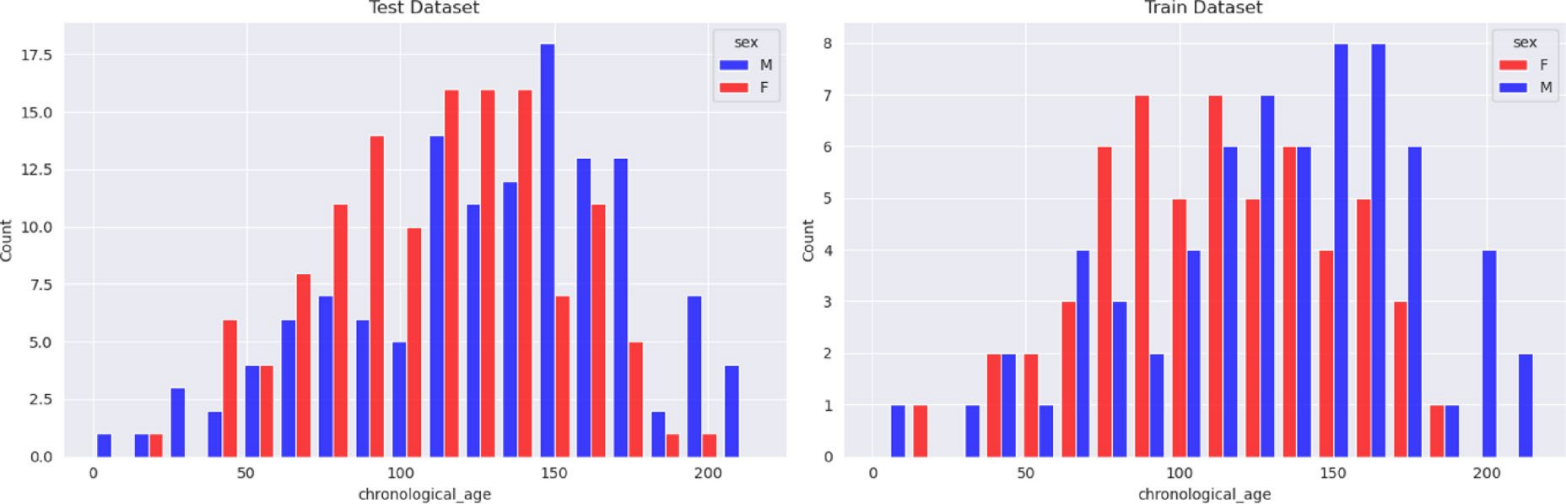
Age distribution in the training (right) and test (left) sets for males and females.

To overcome the systematic over- or underestimation of BA observed in previous studies using other AI methods on generalization across populations, we created a new version of *Deeplasia*, named *Deeplasia-GE*, which is aligned with reference ratings provided by local clinicians and, thus, calibrated to the Georgian population. We hereby fit simple sex-specific linear regression models using a held-out training set (63 males, 58 females, Fig. 2) without retraining *Deeplasia*’s core deep-learning model.

The resulting regression parameters for females were estimated as slope = 1.032 (95% confidence interval, CI: [0.990, 1.073]) and intercept = -6.532 months (95% CI: [-11.512, 1.551]), while for males, the slope was 1.040 (95% CI: [0.999, 1.081]) and the intercept was − 9.860 months (95% CI: [-15.62, -4.10]).

**Fig. 3:**
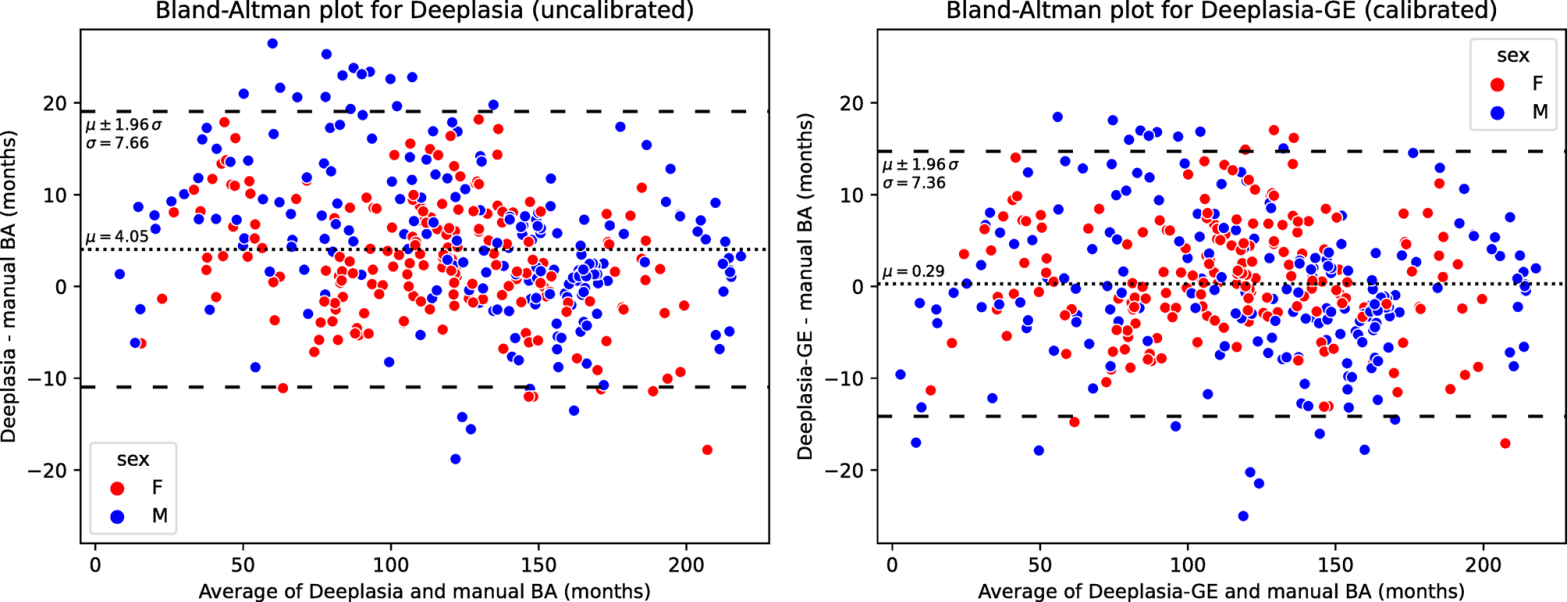
Bland-Altman plots showing the agreement of the average of seven manual bone age ratings in the test set (*n*=260) with *Deeplasia* (uncalibrated version, left) and *Deeplasia-GE* (calibrated version, right). The dotted and dashed lines indicate the average ($\mu$, months) difference with the 95% confidence interval (CI, $\mu \pm 1.96\sigma$ ). Note that 92.3% and 93.9% of cases fall within the CI.

We then compared *Deeplasia* (uncalibrated) and *Deeplasia-GE* (calibrated) to the consensus manual reference rating in the independent test set (126 females, 134 males, Fig. 2) to verify the generalizability of the learned calibration and to estimate the expected real-world performance for Georgian children with respect to local clinicians. The effect of calibration is visualized in the Bland-Altman plots in Fig. 3, whereas the numeric accuracy metrics are provided in Table 1. We see that the calibration reduces the assessed BA in both sexes, with a stronger correction for boys and a decreasing effect of the calibration with increasing age.

**Table 1:** Performance of the uncalibrated base (*Deeplasia*) and the Georgia-specific calibrated version (*Deeplasia-GE*) on the test set of the Georgian bone age dataset. Previous results for the performance in the RSNA, DHA, and GDBD datasets^5^ are provided as a reference. DHA: Los Angeles digital hand atlas, GDBD: German dysplastic bone dataset. MAD: mean absolute difference, RMSE: root mean squared error, RSNA: radiological society of North america. Lower MAD and RMSE indicate higher accuracy. ^b^Estimated range for the accuracies of the assessed single raters.

Dataset	No. Ref. Ratings	*n*	Deeplasia (months)		Inter-rater (months)	
			MAD	RMSE	MAD	RMSE
Georgian	7	260	6.6 (base)	8.8 ([8.1, 9.6]) (base)	7.9	10.6
Georgian	7	260	**5.7** (calibrated)	**7.4 ([6.8, 8.1])** (calibrated)		
RSNA^11^	6	200	3.9	5.1 (4.7, 5.7])	4.8–7.0^b^	-
DHA^23^	2	1383	5.8	7.7 ([7.4, 8.0])	4.4	7.0
GDBD^5^	2	702	6.0	7.7 ([7.3, 8.1])	9.5	12.8

For the uncalibrated version of *Deeplasia*, the mean absolute difference (MAD) was 6.57 months, the root mean squared error (RSME) was 8.76 months (95% CI : [8.06, 9.58]), and the 1-year accuracy was 87.7%. Thus, *Deeplasia*’s accuracy is reduced compared to previous results on other ethnicities, even though the study on Georgian children had a higher number of reference raters, which should result in a more reliable reference rating and, thus, higher assessed performance. When analyzing the calibration (Fig. 3), we observed that the default version of *Deeplasia*, on average, overestimates the BAs in the assessed Georgian cohort with a signed mean difference (SMD) of 2.85 (95% CI : [1.68, 4.01]) months and 5.35 (95% CI : [3.90, 6.81]) for female and male patients, respectively. Nevertheless, *Deeplasia’s* MAD and RMSE are lower than the average inter-rater discrepancy (Table 1), whereby *Deeplasia’s* BA prediction was more accurate than five out of seven raters (Table 2). The intraclass correlation (ICC) between *Deeplasia* and the consensus manual BA was 0.9930 (95% CI: [0.99, 1.00]).

**Table 2:** Performance of individual raters. The performance of *Deeplasia* and *Deeplasia-GE* is compared against each of the seven individual raters to the consensus bone ages established by the remaining six raters. Metrics where the automatic bone age assessment is more accurate than the manual assessment are marked in bold.

	MAD			RMSE		
left out rater	manual	Deeplasia	Deeplasia-GE	manual	Deeplasia	Deeplasia-GE
I	6.4	7.0	**6.0**	8.4 [7.8, 9.2]	**9.2 [8.5**,** 10.1**]	**7.8 [7.2**,** 8.5]**
II	7.3	**7.0**	**6.1**	9.5 [8.7, 10.4]	9.2 [8.5, 10.1]	**7.9 [7.3**,** 8.6]**
III	9.7	**6.6**	**5.6**	12.7 [11.7, 13.9]	**8.7 [8.0**,** 9.5]**	**7.3 [6.7**,** 8.0]**
IV	8.6	**6.3**	**5.4**	11.2 [10.3, 12.2]	**8.5 [7.8**,** 9.3]**	**7.1 [6.5**,** 7.8]**
V	6.7	6.8	**5.8**	8.9 [8.2, 9.7]	9.0 [8.3, 9.8]	**7.4 [6.9**,** 8.1]**
VI	7.4	**6.5**	**5.8**	9.8 [9.0, 10.7]	**8.6 [7.9**,** 9.4]**	**7.4 [6.8**,** 8.1]**
VII	9.3	**7.4**	**6.6**	12.0 [11.1, 13.1]	**9.8 [9.1**,** 10.8]**	**8.4 [7.7**,** 9.2]**

The calibrated version, i.e., *Deeplasia-GE*, reduced the MAD to 5.69 months, RMSE to 7.37 months (95% CI: [6.79, 8.06]), and improved the 1-year accuracy to 88.4%. The calibration effectively overcomes BA overestimation in the Georgian population, almost nullifying the SMD to -0.03 (95% CI: [-1.18, 1.11]) months and 0.58 (95% CI: [-0.81, 1.97]) months for girls and boys, respectively. This finding validates that the learned regression generalizes to the test set.

The Bland-Altman analysis (Fig. 3) further confirmed the improved agreement of *Deeplasia-GE* with the ensemble of reference raters. Consequently, *Deeplasia-GE* is more accurate than all 7 individual raters (Table 2), and the ICC improved to 0.9939 (95% CI: [0.99, 1.00]).

**Fig. 4:**
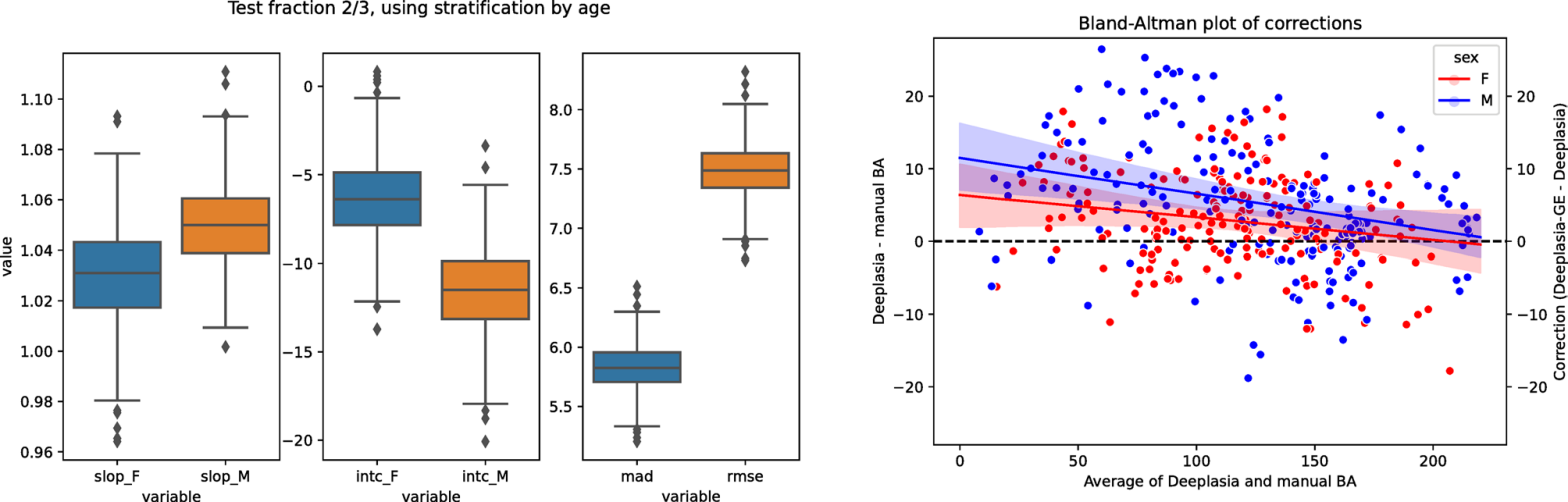
Bootstrap re-partitioning (*n* = 1000) into calibration (⅓) and test (⅔) sets and its effect. Left: the distribution of sex-specific regression parameters (slope and intercept) obtained from the calibration sets and the resulting mean absolute difference (MAD) and root mean squared error (RMSE) in the test sets. Right: the resulting corrections (i.e. difference between *Deeplasia* and *Deeplasia-GE*) as median and bootstrapped 95% CI. The solid lines indicate the correction derived from the selected test set.

To estimate the robustness of the conducted regression correction, we additionally bootstrapped simulated *n* = 1000 alternative train-test partitions. We present the resulting alternative calibration parameters and their effects in Fig. 4. Independent of the dataset partitioning, the conducted calibration falls within the 95% CI of correction of all models, and the described effects are stable across manifestations of the partitionings. Furthermore, based on the same bootstrapping, we estimate the partitioning-specific 95% CIs of the performance metrics at [5.48, 6.16] months MAD, [7.08, 7.87] months RMSE, and [85.1%, 89.7%] 1-year accuracy. Thus, the measured performance gains of *Deeplasia-GE* over the baseline version, *Deeplasia*, are independent of the partitioning of the dataset.

**Fig. 5:**
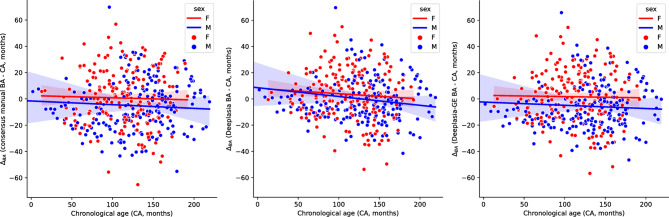
Difference between assessed bone ages (BA) and the chronological age (CA). Left: Consensus bone age (average of seven raters). Center: uncalibrated *Deeplasia*. Right: Calibrated *Deeplasia-GE*. Note that *Deeplasia-GE* better replicated the average deviation of BA from CA than the uncalibrated base version.

As a final analysis, we compared automatic and consensus manual BA estimates to the chronological age (CA, Fig. 5). We observed that with the uncalibrated version, *Deeplasia*, the BA of young boys and girls is on average similar but the BA estimates are slightly higher than CA in young cases and approach CA in older ones. However, for both the manual and the calibrated, *Deeplasia-GE*, estimations the BA in young Georgian boys is, on average, slightly delayed compared to girls. Further, for both manual and *Deeplasia-GE* methods, BA estimations show on average a high agreement with CA. Note that we observe relatively many outliers with a large deviation of the BA from CA with all methods, however, relatively few of these cases have been diagnosed with genetic or growth disorders.

## Discussion

In this work, we created *Deeplasia-GE*, a version of *Deeplasia* calibrated and tailored to the Georgian population. In the allocated test set, we observed that the uncalibrated version showed a systematic overestimation of BA in girls and – even more severely – in boys. Using bootstrapping, we confirmed that this effect is present independent of the exact partitioning. This indicates that the overestimation is systematic within the GBAD. Using these insights, we show that this can be accounted for using simple linear regression models.

The reduced accuracy of *Deeplasia* in the Georgian population is expected due to the observed differences in growth charts of Georgian children compared to Europeans^30^. However, whereas the accuracy (i.e., the numeric values assigned to individual images) for the uncalibrated version of *Deeplasia* is decreased in the Georgian population, the precision (i.e., the ability to discriminate between different groups of BAs/developmental stages or detect deviations from normal maturation patterns) is unaltered by the miscalibration. In turn, limiting the calibration to linear models sustains the precision of *Deeplasia-GE*. Thus, the re-calibration instead of full or partial retraining of the model weights inherently guarantees to sustain *Deeplasia*’s high test-retest reliability^5^ and, thus, applicability in longitudinal applications to *Deeplasia-GE*. Furthermore, Rassmann et al. (2024)^5^ showed that *Deeplasia* has high accuracy for BA assessment of individuals with seven different skeletal disorders (namely achondroplasia, hypochondroplasia, pseudohypoparathyroidism, Noonan, Silver-Russell syndrome, Ullrich-Turner syndromes; SHOX-related short stature, and intrauterine growth restriction). This feature should also be inherited by *Deeplasia-GE*, as it is using the same deep learning image representations. However, further studies are needed to test the applicability of both *Deeplasia* and *Deeplasia-GE* to other skeletal disorders.

As we have a relatively high number of raters, we assume that the averaged manual rating effectively serves as a reliable calibration reference. Yet, we observed that individual raters showed some variation with respect to their individual SMD (see Methods), so the exact calibration might show some degree of bias towards the participating reference raters. Thus, the gains in assessed accuracy might be slightly overestimated compared to BA estimates of other raters.

The lack of a global and uniformly sampled reference dataset hinders the development of a population-agnostic AI for BA assessment. Therefore, given the known differences in growth of children from different ancestries, testing and (when needed) calibrating of AI tools for different populations could be a pragmatic way forward. Furthermore, in this study, we used only 121 training images sampled from the local population, compared to > 12,600 images in the RSNA dataset. Thus, effectively, the proposed method for re-calibration allows for transferring the learned BA assessment from existing, large datasets to a small cohort representative of the target population. In this way, the simple linear re-calibration can help to overcome the general problem of data sparsity due to time-consuming reference ratings when creating BA tools for smaller populations. Yet, regardless of data-efficient re-training, sufficiently large cohorts with reference ratings are required to reliably estimate the population-level performance. We hope that similar testing and (if needed) calibration of existing AI tools will be made for other small populations in the future. As an open-source AI, *Deeplasia* is suitable for this purpose.

## Conclusion

In this work, we tested and calibrated the open-source BA assessment AI, *Deeplasia*, to the Georgian population. We used a re-calibration method to establish an accurate, population-specific BA assessment tool, *Deeplasia-GE*. In addition to inheriting the high precision from *Deeplasia*, we showed that *Deeplasia-GE* is also more accurate than all of our Georgian reference raters. Thus, we suggest *Deeplasia-GE* as a reliable BA assessment AI for Georgian children.

## Method

### Data collection

This is a retrospective study using patients’ hand X-rays. Ethical approval was obtained from, and informed consent was waived by, the institutional review board of the Givi Zhvania Pediatric University Clinic of Tbilisi State Medical University (MES 4 25 0000604228). All methods were performed following the ethical standards of the Helsinki Declaration. We collected a total of 457 hand X-rays from patients aged 0–18 years old. This range covers the full spectrum of skeletal development from infancy through late adolescence, during which ossification progresses and epiphyseal plates gradually fuse with the bone shafts. This process varies across different bones and typically completes by the end of adolescence. Additionally, we include both boys and girls in the study to account for sex-based differences in skeletal maturation, as it is well established that bone fusion tends to occur later in boys than in girls. We manually excluded 22 images due to bad quality or incomplete representation of the regions relevant for BA estimation (carpal and metacarpal bones) and then selected 400 images for BA reference rating.

### Reference rating

The manual BA reference rating was performed using the GP atlas. The images included left and right hands, and we selected the left hand whenever possible. All raters assessed the X-rays individually and without knowledge of the CA on de-identified images. The raters were three radiologists and four endocrinologists.

For 17 images, at least one rater was not able to conduct a BA assessment due to bad image quality or asynchronous BA within the images. Together with another 2 images in which at least one individual BA rating deviated > 30 months from an initial, uncorrected BA average, these images were excluded from the analysis. Hence, 381 images were included in the dataset,19 of these from children with a known genetic disorder.

We established the final consensus reference BA following the approach by Halabi et al. (2019)^11^. In brief, the individual BA ratings were corrected by subtracting each rater’s SMD from the initial, uncorrected consensus BA (range: [-3.07, 1.55] months). Then, we formed a performance-weighted average across raters, where each rater’s weight was proportional to 1/MAD (range: [0.112, 0.171]).

For comparing *Deeplasia* and *Deeplasia-GE* to individual raters, the respective rater was removed, and the consensus was re-calculated using weights obtained from only the remaining raters. We then compared each model version and the held-out rater against the consensus of the remaining raters.

### Bone age prediction and calibration

*Deeplasia* consists of a hand-masking and an ensemble of three deep convolutional neural networks (CNNs) conducting BA estimation as an ensemble. These models were trained on the training set of the 2017 pediatric BA challenge of the RSNA covering an age range of 0–18 years. For details, see Rassmann et al. (2024)^5^.

To obtain the re-calibrated bone age, ${\text{B}}{{\text{A}}_{{\text{Deeplasia-GE}}}}$, linear regression models as.

$${\text{B}}{{\text{A}}_{{\text{Deeplasia-GE}}}}{\text{=}}{\kern 1pt} {\text{slop}}{{\text{e}}_{{\text{sex=m/f}}}}\, \cdot {\text{B}}{{\text{A}}_{{\text{Deeplasia}}}}{\text{+}}{\kern 1pt} {\text{intercep}}{{\text{t}}_{{\text{sex=m/f}}}},$$

where ${\text{B}}{{\text{A}}_{{\text{Deeplasia}}}}$ are the predictions performed by *Deeplasia* in months, and ${\text{slop}}{{\text{e}}_{{\text{sex=m/f}}}}$ and ${\text{intercep}}{{\text{t}}_{{\text{sex=m/f}}}}$ are the sex-specific parameters of calibration.

The regression analysis was conducted using scikit-learn (v1.2.2) in Python (v3.9). We randomly split the data into train and test partitions, stratifying for age and sex and assigning images of children with known genetic disorders to the test set.

### Statistical analysis

For a definition of the performance metrics, see Rassmann et al. (2024)^5^. The 95% CI of the RMSE was computed based on the chi-squared $\left( {{\chi^2}} \right)$ distribution, assuming normally distributed residual errors. Thus, the CIs can be derived as.

$$\left( {\sqrt {\frac{{n \times {\text{RMS}}{{\text{E}}^2}}}{{\chi_{{0.975,n}}^{2}}}} ,\sqrt {\frac{{n \times {\text{RMS}}{{\text{E}}^2}}}{{\chi_{{0.025,n}}^{2}}}} } \right),$$

where $n$ is the sample size, and $\chi_{{0.025,n}}^{2}$ and $\chi_{{0.975,n}}^{2}$ represent the 2.5th and 97.5th percentiles of the ${\chi^2}$ distribution with $n$ degrees of freedom.

We computed the 95% CIs for the SMDs using the standard error of the mean (SEM) and a Student’s t-distribution, deriving the CIs using:

$$\bar {d} \pm {t_{0.975,n - 1}} \times S{E_d},$$

where $\bar {d}$ is the SMD, $S{E_d}=\frac{{{\sigma _d}}}{{\sqrt n }}$ is the standard error, ${\sigma _d}$ is the sample standard deviation, *n* is the sample size, and ${t_{0.975,n - 1}}$ is the critical value from the *t*-distribution with *n* degrees of freedom. We tested for the normality of the signed residuals using the Kolmogorov-Smirnov test. Using normality as the null hypothesis, we found *p* > 0.05 in all tests and, thus, assumed normality.

ICC estimates and their 95% CIs were calculated based on a two-way random effect model against the mean rating (*k* = 7) on the absolute agreement (ICC (2,k)).

All statistical analyses were conducted in Python using the Scipy (v1.13), statsmodel (v0.14), and pingouin (v0.5) packages.

## Data Availability

The datasets used and analysed during the current study can be made available from the corresponding author on reasonable request.
